# Transcriptomics yields valuable information regarding the response mechanisms of Chinese Min pigs infected with PEDV

**DOI:** 10.3389/fvets.2023.1295723

**Published:** 2023-12-11

**Authors:** Huihui Li, Chunxiang Zhou, Meimei Zhang, Na Yuan, Xiaoyu Huang, Jiaojiao Xiang, Lixian Wang, Lijun Shi

**Affiliations:** ^1^Institute of Animal Science, Chinese Academy of Agricultural Sciences, Beijing, China; ^2^Huanghe Science and Technology University, Zhengzhou, China; ^3^Beijing Vica Biotechnology Co., LTD, Beijing, China

**Keywords:** Min pigs, PEDV, transcriptome, WGCNA, transcript factor

## Abstract

Porcine epidemic diarrhea virus (PEDV) causes porcine epidemic diarrhea (PED), a highly infectious disease, which has resulted in huge economic losses for the pig industry. To date, the pathogenic and immune response mechanism was not particularly clear. The purpose of this study was to investigate the pathogenic and immune responses of pigs infected with PEDV.In this study, 12 Min pigs were randomly selected without taking colostrum. At 3 days old, eight piglets were infected with 1 mL of PEDV solution (10 TCID50/ml), and the remaining four piglets were handled by 1 mL of 0.9% normal saline. Within the age of 7 days old, four piglets died and were considered as the death group. Correspondingly, four alive individuals were classified into the resistance group. Tissues of the duodenum, jejunum, ileum, colon, cecum, and rectum of piglets in the three groups were collected to measure the PEDV content. Additionally, the jejunum was used for the measurements and analyses of Hematoxylin-eosinstaining (HE), immunohistochemical sections, and transcriptomics. The phenotypes of Min piglets infected with PEDV showed that the viral copy numbers and jejunal damage had significant differences between the death and resistance groups. We also observed the transcriptome of the jejunum, and the differentially expressed (DE) analysis observed 6,585 DE protein-coding genes (PCGs), 3,188 DE long non-coding RNAs (lncRNAs), and 350 DE microRNAs (miRNAs), which were mainly involved in immune response and metabolic pathways. Furthermore, the specific expressed molecules for each group were identified, and 97 PCGs,108 lncRNAs, and 51 miRNAs were included in the ceRNA-regulated networks. By weighted gene co-expression network analysis (WGCNA) and transcription factor (TF) prediction, 27 significant modules and 32 significant motifs (*E*-value < 0.05) annotated with 519 TFs were detected. Of these TFs, 53 were DE PCGs. In summary, the promising key PCGs, lncRNAs, and miRNAs related to the pathogenic and immunological response of pigs infected with PEDV were detected and provided new insights into the pathogenesis of PEDV.

## 1 Introduction

Porcine epidemic diarrhea (PED), induced by the porcine epidemic diarrhea virus (PEDV), is a disease with high contagion. PEDV is an enveloped, single-stranded, positive-sense RNA virus that targets the small intestine, causing villi atrophy and vacuolation (1, 2). Piglets infected with PEDV experience watery diarrhea due to the interruption of digestion and absorption of nutrients (3). Currently, the most crucial approach to managing PEDV is through vaccination; however, PEDV is prone to mutation to intensify the virulence (1). Over the past three decades, PED has been widespread worldwide and caused huge economic losses in the pig industry. Various coronaviruses emerged due to cross-species transmission among humans and domestic animals, and the cross-species transmission of PEDV is also possible (4). Additionally, PEDV is airborne, pollutes the environment, predisposes large pig populations to infection, and poses a potential threat to livestock farming (5).

Uncovering the genetic mechanism of host resistance against PEDV is crucial. Based on the porcine 80 K SNP Chip, F. Bertolini et al. calculated the Fst value between dead and recovered groups of pigs infected with PEDV and identified seven highly divergent windows including 152 genes (6). According to the transcriptome of Vero cells, IPEC-J2 cells, and small intestinal mucosa of piglets infected with PEDV, genes involved in TNF signaling, inflammatory response, cytokine receptor interaction, and other immune-related pathways were identified to play important roles in response to PEDV infection, such as *IFIT1, MX2, TRIM25, CXCL2*, and *IL-1β* (7–9). Long non-coding RNAs (lncRNAs) might play important roles in host antiviral responses, for example, the lncRNA-9606 involved in the intestinal immune network for IgA production was upregulated 400-fold in the ileum infected with PEDV and at least 300-fold in IPEC-J2 cells infected with PEDV (10).

Min pigs have the characteristics of excellent meat quality and stress and disease resistance, while the response mechanism of Min pigs infected with PEDV is lacking. In this study, we created a Min pig population infected with PEDV and divided the individuals into death, resistance, and control groups. We constructed the PEDV standard curve, measured the phenotypes of pigs in each group, and conducted transcriptome analysis of the jejunum tissues to identify the differentially expressed (DE) protein-coding genes (PCGs), lncRNAs, and miRNAs across the three groups. Furthermore, we performed weighted gene co-expression network analysis (WGCNA), constructed ceRNA regulatory networks, and predicted the transcription factors (TFs) bound with DE PCGs to identify the key molecules involved in the pathogenic and immune response mechanism. The study might be good for understanding the molecular mechanisms underlying the response of pigs infected with PEDV and provides a biological basis for the process of PEDV infection.

## 2 Materials and methods

### 2.1 Animals and viruses

**Figure 1:**
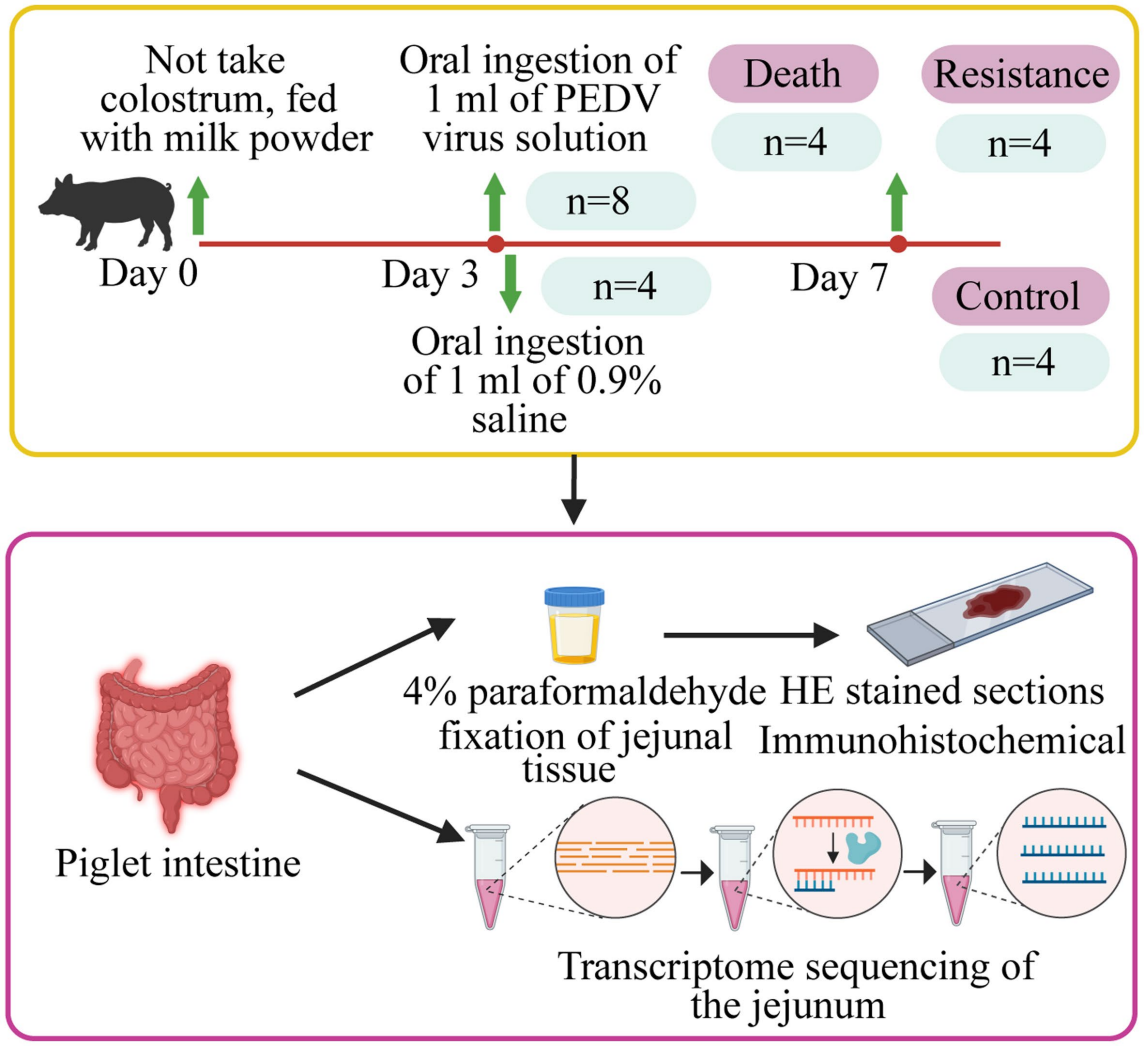
Details of the study design. Piglets were fed with milk powder until 3 days of age. Eight pigs were randomly selected for infection and four pigs were selected as control piglets. The jejunal tissues of infected piglets that died, resistant piglets, and control piglets were collected to make HE-stained sections, immunohistochemical analysis, and whole transcriptome sequencing.

In this study, we randomly selected 12 Min piglets from the Changping Pig Breeding Farm of the Institute of Animal Science, Chinese Academy of Agricultural Sciences (Beijing, China), and individually raised them in a cardboard box with a constant temperature of 35°C. These piglets did not take colostrum and were fed with BaoBaoLe milk [Centree Bio-Tech (Wuhan) Co., LTD, Wuhan, China]. At 3 days old, eight piglets were infected with 1 mL of PEDV solution (10 TCID_50_/ml), which was a G2a mutant virus isolated from the jejunum of pigs in Beijing Liuma Pig Raising Technology Co., LTD in 2014 (Shunyi, Beijing, China). The remaining four piglets, as the control group, were handled by 1 mL of 0.9% normal saline (Beijing Epsilon Biotechnology Co., LTD, Beijing, China; Figure 1).

### 2.2 Tissue collection

After infection, we monitored the time of diarrhea and vomiting in piglets. At 24 h after infection, we collected the feces of each pig and detected whether PED occurred using an Anigen Rapid PED Ag Test Kit (Anigen, Korea). If the viral load was higher than 10^5^ TCID_50_/ml, two colored lines would appear on the test paper, implying a PEDV-positive infection. Within the age of 7 days old, four min pigs died and were considered as the death group. Correspondingly, four alive individuals were classified into the resistance group. The pigs in the resistance and control groups were injected with xylazine hydrochloride injection (0.2 mL/kg; Beijing Lab Anim Tech Develop Co., LTD, Beijing) for anesthesia and then were dissected. Tissues of the duodenum, jejunum, ileum, colon, cecum, and rectum of piglets in the three groups were washed with 0.9% saline (Beijing Epsilon Biotechnology Co., LTD, Beijing, China), collected, and then stored in liquid nitrogen for measurement of phenotype and transcriptome. In addition, the 1 cm^3^ jejunum from each pig was fixed in 4% paraformaldehyde (Beijing Biotopped Technology Co., LTD, Beijing, China) for paraffin sectioning.

### 2.3 Construction of PEDV standard curve and evaluation of PEDV content

The viral nucleic acid of each intestine was extracted using a MagaBio Plus Viral DNA/RNA Purification Kit (Hangzhou Biotechnology Co., Ltd., Hangzhou, China). It was reversely transcribed to the cDNA using a TaKaRa Reverse Transcription Kit (TaKaRa Bio, Japan). The primers (Supplementary Table S1) of the N gene of the PEDV were designed by Primer 3 and synthesized in Shenzhen Huada Gene Co., Ltd. (Shenzhen, China). The qRT-PCR amplifications were carried out on an Applied Biosystems PCR instrument (Thermo Fisher Scientific, United States) with the following procedure: (a) 95°C for 5 min; (b) 95°C for 30 s, 56°C for 40 s, 72°C for 1 min, 34 cycles; (c) 72°C for 10 min. The amplified N gene fragment was purified by gel recovery, and the purified product was ligated to pCE2 TA/Blunt-Zero (Novozymes, Nanjing, China) vector for 5 min at room temperature (20–37°C). In total, 10 μL of the above vector was introduced into DH5α receptor cells (Novozymes, Nanjing, China), mixed with 900 μL of LB liquid medium (without antibiotics), and then shaken for 1 h at 37°C with a shaker at 200 rpm for recovery. Overall, 900 μL of the supernatant was discarded, and the bacteria were resuspended in LB liquid medium and spread evenly on LB solid medium plates that showed ampicillin resistance. Colonies formed by individual bacteria were picked out and placed in the liquid LB medium for 10 h. The bacterial solution in centrifuge tubes was sent to the laboratory to test for successful ligation of N gene fragments of PEDV. DNA was extracted from the positive plasmids, and the concentration was determined by calculating the number of plasmid copies per 1 μL of standard plasmid based on the following formula and the seven gradient dilutions from 10^2^ to 10^8^:


$$\begin{array}{l} \mathrm{Number}\;\mathrm{of}\;\mathrm{copies}\left(\mathrm{Copies}/\mathrm{\mu l}\right)=\\ \frac{6.02\times 10^{23}\left(\mathrm{Number}\;\mathrm{of}\;\mathrm{copies}/\mathrm{mol}\right)\times \mathrm{Plasmid}\;\mathrm{concentration}\left(\mathrm{ng}/\mathrm{\mu l}\right)\times 10^{-9}}{\mathrm{Number}\;\mathrm{of}\;\mathrm{plasmid}\;\mathrm{bases}\times 660\;\mathrm{dalton}/\mathrm{bp}} \end{array}$$

The qRT-PCR reactions were performed with the above gradient dilutions of DNA on an ABI Q7 Flex 384-well fluorescent quantitative PCR instrument with the following procedure: (a) 30 s at 95°C; (b) 40 cycles of 5 s at 95°C and 34 s at 60°C; (c) 15 s at 95°C and 1 min at 60°C and 95°C for 15 s. In total, three replicate wells per DNA spot were implied. Standard curves were plotted according to Ct values vs. plasmid copy number. The PEDV cDNAs from six intestinal segment tissues were quantified using the procedure described above and substituted into the standard curve to calculate the viral copy number. The differences in the viral copy numbers between the death and resistance groups were analyzed by the T-test, in which the significant threshold was *p-*value <0.05.

### 2.4 Pathological examination

We fixed 1 cm × 1 cm × 1 cm jejunum of each piglet in 4% paraformaldehyde (Beijing Biotopped Technology Co., LTD, Beijing, China) to obtain the paraffin sections. According to the reported procedure mentioned in the reference (11), each fixed jejunum was cut and stained with Hematoxylin-eosinstaining (HE). Immunohistochemistry (IHC) was involved in dewaxing paraffin-embedded tissue slices and heating them in a pressure cooker. Antigen retrieval was performed on the slices using a citrate antigen retrieval solution (Beyotime Biotech. Inc., Shanghai, China) in a microwave oven. Endogenous peroxidase activity was blocked using 3% H_2_O_2_. Sections were incubated with normal goat serum buffer for 20 min and then with a mouse anti-PEDV N mAb (diluted 1:200) for 1 h at room temperature. Sections were then incubated with a secondary antibody before a DAB Horseradish Peroxidase Color Development Kit (JK GREEN Tech INC., Beijing, China) was used to develop the color reaction. The cell nucleus was re-stained with Mayer’s hematoxylin for 1 min. The brown staining of PEDV on intestinal villous epithelial cells was classified as positive staining. Pathological changes in the intestinal tissue samples were observed under a Leica DM300 microscope (Leica Microsystems, Wetzlar, Germany). In addition, we used ImageJ software for statistical analysis of the proportion of positive signal area.

### 2.5 Library construction and RNA sequencing

The total RNA of the jejunum from each piglet was extracted using a TRlzol reagent kit (Invitrogen, United States), according to the manufacturer’s instructions. The RNA concentration and purity were, respectively, measured using a Qubit^®^ RNA Assay Kit in Qubit^®^ 2.0 Fluorometer (Life Technologies, CA, United States) and a NanoPhotometer^®^ spectrophotometer (IMPLEN, CA, United States). The RNA integrity was checked using an RNA 6000 Nano Assay Kit (Agilent Technologies, CA, United States) on a Bioanalyzer 2,100 system (Agilent Technologies, CA, United States).

For mRNA and lncRNA, 3 μg RNA of each sample was used as input material for the RNA library preparation. Strand-specific libraries were constructed by the ribosome RNA removal method, following the manufacturer’s recommendations for the NEBNext^®^ Ultra^TM^ RNA Library Prep Kit for Illumina^®^ (NEB, United States). RNA was broken into short fragments of 250–300 bp, which were used as templates to synthesize cDNA. Then, the PCR amplifications were performed, and their products were purified (AMPure XP system). The library quality was assessed on an Agilent Bioanalyzer 2,100 system. Finally, the library preparations were sequenced on a Novaseq 6,000 platform (Illumina, NEB, United States), and 150 bp strand-specific paired-end reads were generated.

For the small RNA, a Small RNA Sample Pre Kit (Illumina, NEB, United States) was used to construct the library. Small RNA was directly spliced at both ends and then reversely transcribed to cDNA. After PCR amplification, the target DNA fragments were separated by PAGE gel electrophoresis, and the cDNA library was obtained by cutting and recovering the gel. Library quality was assessed on an Agilent Bioanalyzer 2,100 system using high-sensitivity DNA chips. The library preparations were sequenced on a Novaseq 6,000 platform, and 50 bp single-end reads were generated.

### 2.6 Transcriptome analysis

The raw reads containing adapter, ploy-N, and low-quality reads were removed to obtain the clean reads, which were aligned to the Sscrofa11.1 genome assembly^1^ by HISAT2 (12). For mRNAs and known lncRNAs, StringTie (13) was used to assemble and quantify the expressed genes and transcripts. For novel lncRNAs, we identified the following criteria: exon number ≥ 2; transcription length > 200 bp; open reading frame (ORF) ≤ 300 bp; expressed in at least two samples; and non-overlap with the annotated exons in the reference genome. We used three programs, namely, CPC2 (14), PLEK (15), and CNCI (16), with default parameters to assess the coding potential of transcripts. StringTie (13) was also used to quantify the expressed transcripts.

We mapped the clean reads of small RNA to the Sscrofa11.1 reference genomes by Bowtie (17) and identified known miRNAs using miRDeep2 (18) based on miRbase.^2^ We used an miRDeep2 core module, miRDeep2.pl., to predict the novel miRNAs according to the characteristic hairpin structure of miRNA precursors, in which the identified novel miRNAs were expressed in at least two samples.

PCGs and lncRNAs were included in the differential expression analysis only if their FPKM ≥ 0.01 in at least four samples, and for miRNAs, only those with CPM ≥ 0.01 in at least four samples were considered for analysis. Additionally, we performed principal component analysis (PCA) with the package ggplot2 in R to evaluate sample repeatability and grouping.

### 2.7 Differentially expressed analyses and target gene prediction

We used the DESeq2 package in R to identify DE PCGs, lncRNAs, and miRNAs between any two groups (|log2FoldChange | > 1 and adjusted *p-*value < 0.05). In addition, we detected the specifically expressed molecule according to the high upregulated DE fold (≥36).

For the DE lncRNAs and miRNAs, we predicted their target genes. The PCGs within 100 kb upstream and downstream regions of DE lncRNAs were predicted to be the cis-targets by BEDTools (19). The PCGs with high Pearson correlation coefficients >0.95 and *p-*value <0.05 between DE lncRNAs were considered as trans-targets. The PCGs of DE miRNAs were predicted using miRanda (Energy < −40) (20) and TargetScan (Score > 80) (20).

### 2.8 Construction of competing endogenous RNA regulatory network

We predicted the interaction of lncRNAs with miRNAs using miRanda and calculated the Pearson correlation (cor) coefficients between relationship pairs with the Hmisc package of R software, where the pairs with Energy < −40, Score > 180, and |cor| > 0.9 were reserved. The ceRNA network was visualized by Cytoscape software (21). In the ceRNA network, all the PCGs, lncRNAs, and miRNAs were DE molecules.

### 2.9 Transcription factor binding motif analysis

Using MEME Suite,^3^ we researched enrichments of the TFs bound in the promoters of DE PCGs. Specifically, we utilized the BioMart tool from the Ensembl database^4^ to extract 2000 bp sequences upstream of the DE PCGs. Subsequently, the STREME tool was employed to enrich for motifs of width 8-15 from these sequences (*E*-value <0.05). Furthermore, the discovered motifs were compared with the JASPAR CORE vertebrates database (2022) using the Tomtom tool to obtain exact motif annotations (*E*-value <10). Using the Find Individual Motif Occurrences (FIMO) tool, we researched the DE PCGs matched to TFs, in which the threshold was *p*-value <1E˗04.

### 2.10 Weighted correlation network analysis

We used the “WGCNA” package in R to construct the molecule co-expression networks including PCGs, lncRNAs, and miRNAs. In this study, we used the soft-threshold β based on scale-free R2 = 0.90 and transformed the adjacency matrix into a topological overlap matrix (TOM).

### 2.11 Functional annotation

To annotate the functions of key PCGs, lncRNAs, and miRNAs, we performed functional enrichment analysis using the David online website (22), where the GO terms and KEGG pathways with *p*-values <0.05 were significant.

### 2.12 Quantitative real-time PCR

For validating the repeatability and reproducibility of the RNA sequencing data, qRT-PCR was performed on five DE PCGs, five DE lncRNAs, and five DE miRNAs that were randomly selected. We reversely transcribed the total RNA used for transcriptomics into cDNA using a PrimeScript^™^ RT reagent kit with gDNA Eraser (TaKaRa Bio, Kusatsu, Japan). The expression of PCGs and lncRNAs was normalized against the housekeeping gene *GAPDH*, and the expression of miRNAs was normalized against U6 snoRNA. Primers of PCGs/lncRNAs and miRNAs were designed using Primer 3 and miRprimer, respectively. qRT-PCR was conducted in triplicate using TB Green^™^ Premix Ex Taq^™^ (Tli RNase H Plus) on QuantStudio^™^ 7 Flex (Applied Biosystems, Carlsbad, United States) with the following program: 95°C for 30 s; 95°C for 5 s, 60°C for 34 s, 40 cycles; 95°C for 15 s, 60°C for 1 min, 95°C for 15 s.

## 3 Results

### 3.1 Construction of PEDV standard curve and PEDV measurement

**Figure 2:**
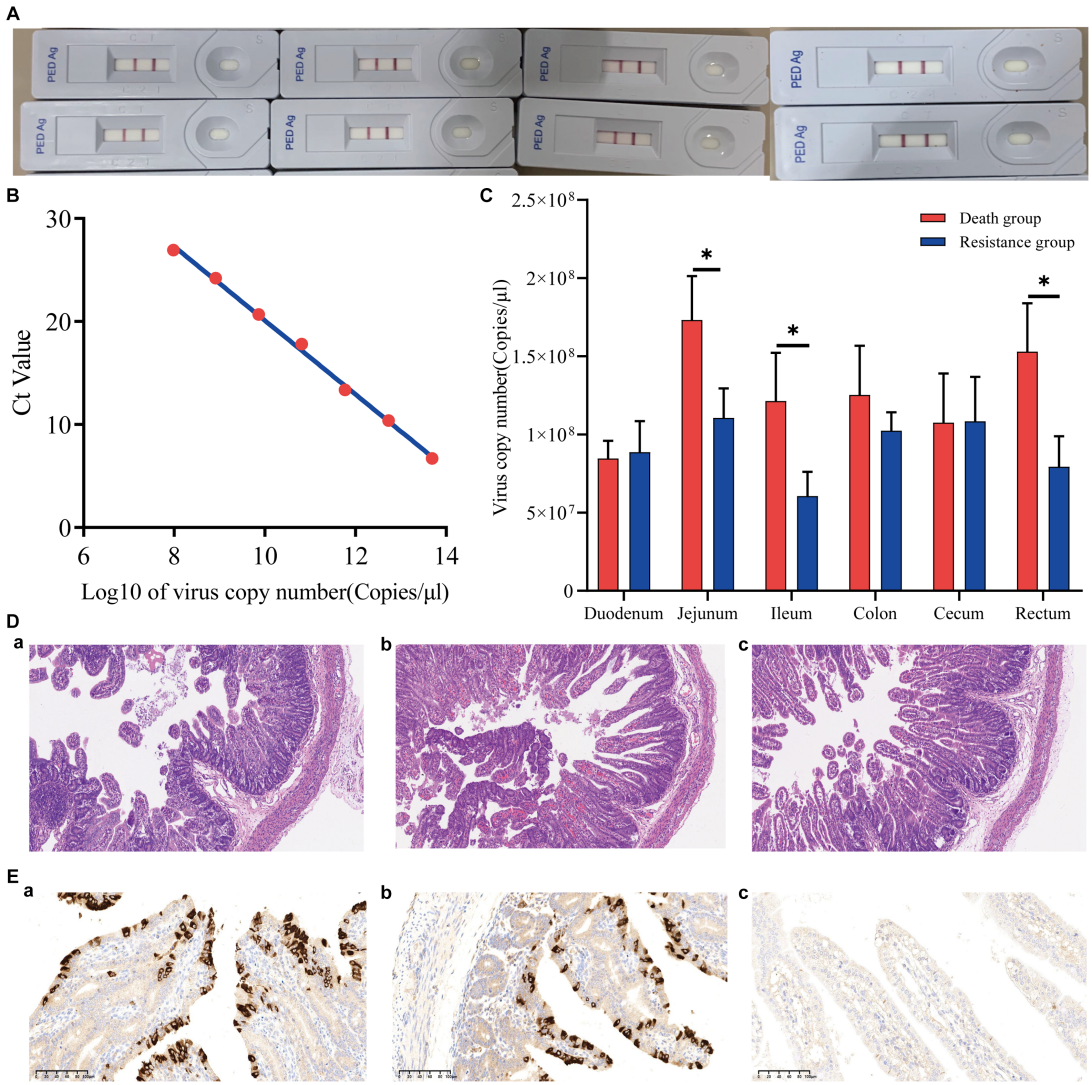
Phenotypic results of piglets infected with PEDV. **(A)** Positive results for eight piglets infected with PEDV. **(B)** PEDV virus copy number standard curve. The Ct value was obtained by qRT-PCR, and the Log10 of the virus copy number was the horizontal coordinate. **(C)** Virus copy number in six intestinal segments of Min pigs. *indicates *p*-value <0.05. **(D)** Hematoxylin–eosin staining (HE) slices of the jejunum of Min pigs with a microscope magnification of 10 × 10. **(A–C)** are death, resistance, and control groups, respectively. **(E)** Immunohistochemical (IHC) slices of the jejunum of Min pigs with a microscope magnification of 10 × 20. **(A–C)** are death, resistance, and control groups, respectively.

Eight piglets were tested with the Anigen Rapid PED Ag Test Kit, and all results were positive (Figure 2A). The PEDV standard plasmid was diluted from 10^2^ to 10^8^ in a 10-fold gradient, and three replicates were set up for each gradient. qRT-PCR was performed, and the corresponding standard curve was plotted (Figure 2B) as follows:


y=−3.5881x+55.983

where x represents Log10 of the virus copy number and y represents the Ct value obtained by qRT-PCR. The R^2^ of the standard curve was 0.9975.

The viral copy numbers of PEDV in the duodenum, jejunum, ileum, colon, cecum, and rectum tissues of Min pigs were calculated according to the standard curve (Figure 2C), and the virus copy numbers of the jejunum, ileum, and rectum in piglets of the death group were significantly higher than those of the resistant group (Figure 2C; *p* < 0.05). The jejunum had the highest viral copy number compared to the intestines of both death and resistant pigs (Figure 2C). For the control piglets, we conducted qRT-PCR to measure their virus copy numbers in the intestines, and no Ct value was presented that indicated no PEDV infection.

### 3.2 Assessment of jejunal injury

We analyzed the pathological changes in the jejunal tissue (Figure 2D) and found that the villi length in death (243.67 ± 8.96 μm) and resistant piglets (336.67 ± 6.80 μm) was significantly shorter (*p* < 0.05) than that in control piglets (432.33 ± 57.71 μm). In addition, the villi length in death piglets was significantly different from the resistant piglets (*p* < 0.05). The percentage of the villus length to crypt depth decreased from 5.04 ± 1.27 (control pigs) to 3.97 ± 0.69 (resistant pigs) and 3.17 ± 0.59 (death pigs; *p* > 0.05). These findings implied that PEDV causes intestinal damage in piglets and that more severe intestinal damage might result in earlier death. The IHC analysis revealed that PEDV-specific antigens (brown stain) were mainly distributed in the jejunum villus epithelial cells of challenged piglets. We selected a photo from a 10 × 20 field of view under a microscope and calculated the proportion of positive signal area using ImageJ. It was found that the proportion of the dead group (9.58%) was higher than that of the resistant group (6.25%). In addition, the control group showed a negative result (Figure 2E).

### 3.3 Identification of differentially expressed transcriptome molecules

**Figure 3:**
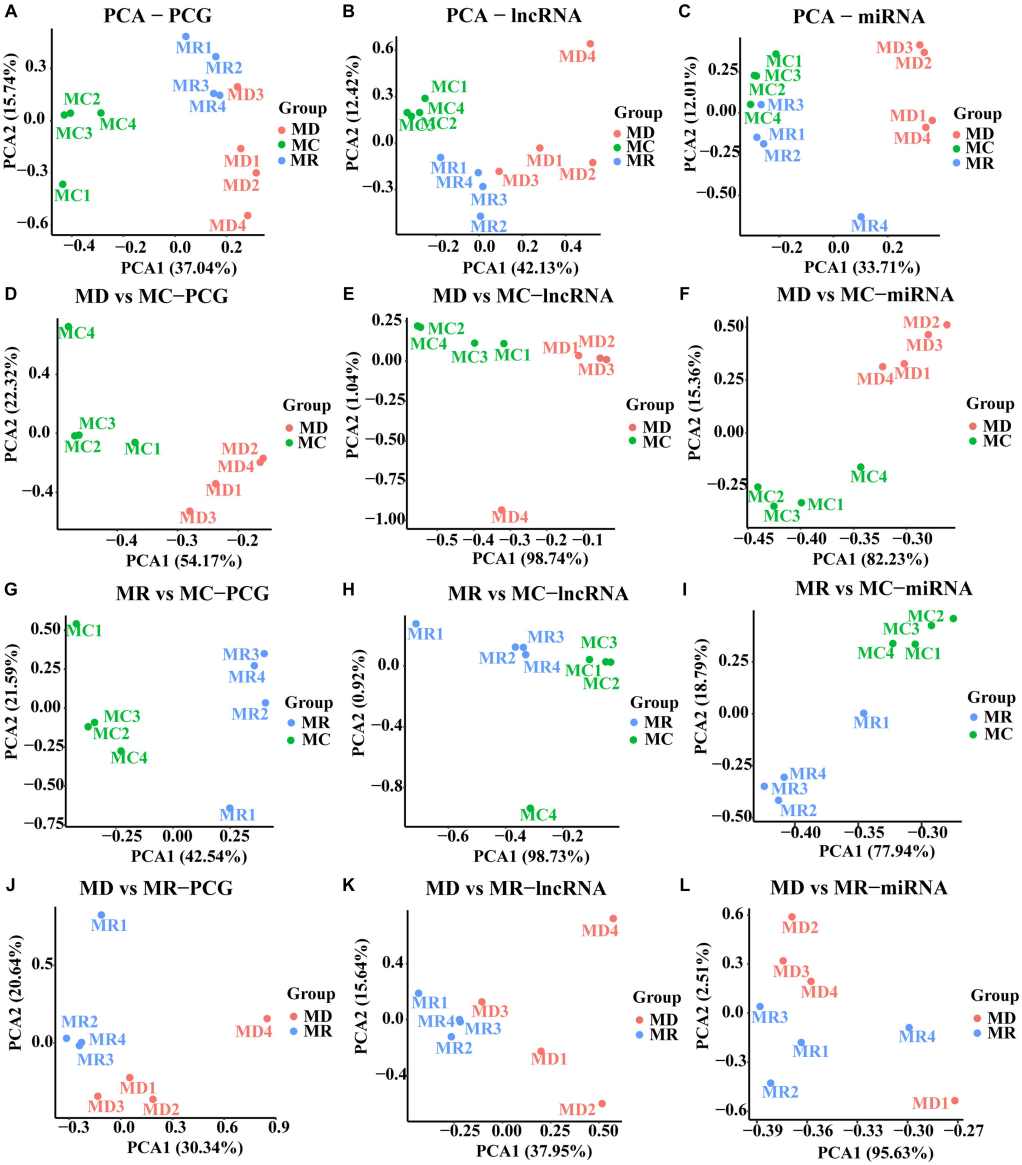
Principal component analysis (PCA) plots of PCGs, lncRNAs, and miRNAs. **(A–C)** PCA analyses performed with the expression of PCG, lncRNA, and miRNA in the three groups, respectively. **(D–E)** PCA analyses performed with the expressions of PCG, lncRNA and miRNA in the death and control groups, respectively. **(G–I)** PCA analyses performed with the expression of PCG, lncRNA and miRNA in the death and control groups, respectively. **(J–L)** are PCA analyses performed with the expression of PCG, lncRNA and miRNA in the death and control groups, respectively. MC: Min piglets of the control group. MD: Min piglets of the death group.MR: Min piglets of the resistance group.

After quality control, we obtained 54,580,522 clean data and identified 16,155 PCGs, 8,386 lncRNAs (3,114 known and 5,272 novel), and 890 miRNAs (344 known and 546 novel; Supplementary Table S1). Furthermore, we conducted PCA based on PCGs, lncRNAs, and miRNAs, which showed excellent sample repeatability and grouping (Figure 3).

**Figure 4:**
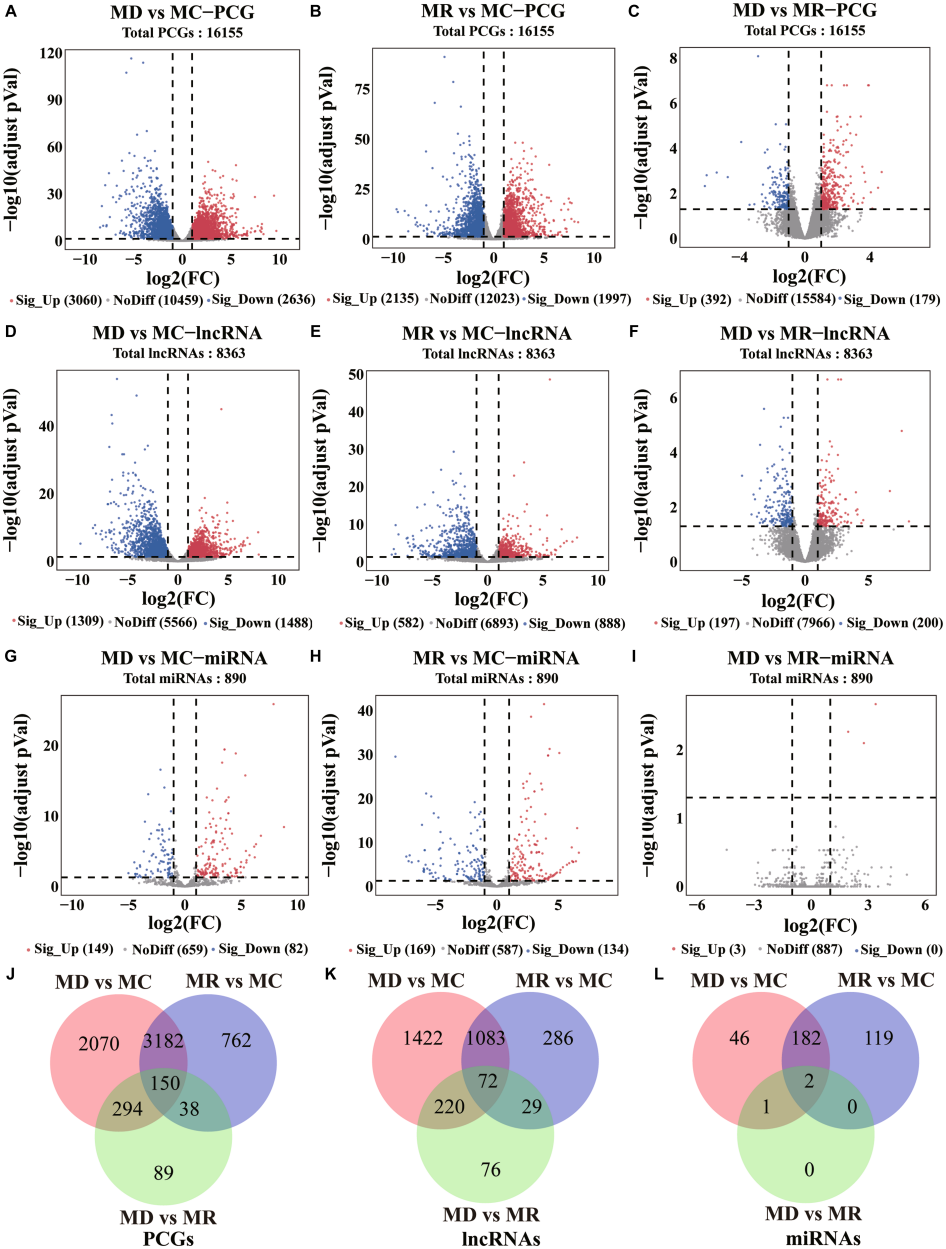
Differential expression analysis results of PCGs, lncRNAs, and miRNAs. MC: Min piglets of the control group. MD: Min piglets of the death group. MR: Min piglets of the resistance group. **(A–I)** Volcano maps of difference analyses of PCGs, lncRNAs, and miRNAs. Sig_up: Significant upregulation. NoDiff: No difference. Sig_Down: Significant downregulation. The number indicates the number of molecules. **(J–L)** Overlap of differential PCGs, lncRNAs, and miRNAs identified in the three compared groups. The number indicates the number of molecules.

Using differential expression analysis, we identified 6,585 DE PCGs (|log2FoldChange | > 1 and adjusted *p-value* < 0.05; Figures 4A–C, Supplementary Table S2), including 5,696 (3,060 upregulated and 2,636 downregulated) in death vs. control piglets, 4,132 (2,135 upregulated and 1,997 downregulated) in resistant vs. control piglets, and 571 (392 upregulated and 179 downregulated genes) in the death group vs. resistant piglets. In addition, 3,188 DE lncRNAs were found (|log2FoldChange | > 1 and adjusted *p-value* < 0.05; Figures 4D–F, Supplementary Table S2), including 2,797 (1,309 upregulated and 1,470 downregulated) in death vs. control piglets, 1,470 (582 upregulated and 888 downregulated) in resistant vs. control piglets, and 397 (192 upregulated and 200 downregulated genes) in death vs. resistant piglets. For miRNAs, 350 DE miRNAs were detected (|log2FoldChange | > 1 and adjusted *p-value* < 0.05; Figures 4G–I, Supplementary Table S2), containing 231 (149 upregulated and 82 downregulated) in the death group vs. control piglets, 303 (169 upregulated and 134 downregulated) in resistant vs. control piglets, and 3 upregulated miRNAs in death vs. resistant piglets. In total, 150 DE PCGs, 72 DE lncRNAs, and 2 DE miRNAs were analyzed in the three comparison groups (Figures 4J–L, Supplementary Table S2).

### 3.4 Target gene prediction and functional annotation

A total of 9,361 target genes were predicted for 2,925 DE lncRNAs, of which 5,012 (Supplementary Table S2) were DE PCGs (4,809 in the death vs. control piglets, 4,373 in the resistance vs. control piglets, and 2,151 in the death vs. resistance piglets). In addition, 341 DE miRNAs obtained 7,691 target genes, of which 2,609 (Supplementary Table S3) were DE PCGs (2,086 in the death vs. control piglets, 2,486 in the resistance vs. control piglets, and 3 in the death vs. resistance piglets).

We annotated the function of the DE PCGs, lncRNAs, and miRNAs and detected 243 significant KEGGs and 1,599 significant GO terms. In detail, the upregulated molecules in death vs. control and resistance vs. control piglets were mainly involved in environmental information processes and systemic pathways related to immunity, such as chemokine, JAK–STAT, TNF, FoxO, IL-17, and MAPK signaling pathways. Correspondingly, the downregulated molecules were mainly associated with metabolic pathways, such as cholesterol, tryptophan, and carbon metabolisms (Supplementary Figures S1–S6).

**Figure 5:**
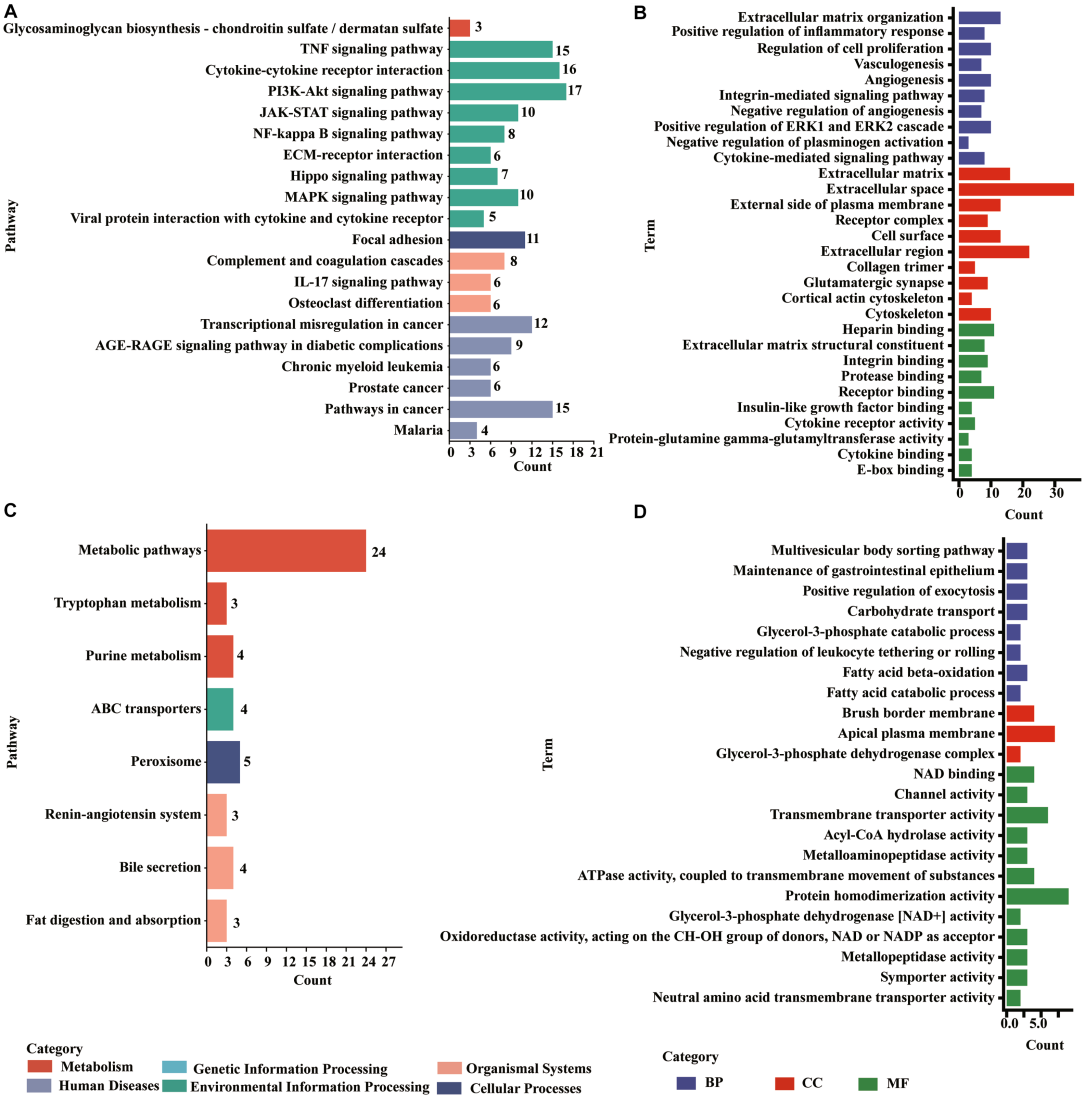
Enrichments of DE PCGs in the Min death (MD) vs. Min resistance (MR) groups. **(A)** KEGG pathway enriched by DE PCGs with significant upregulation expression in MD vs. MR. **(B)** GO entries enriched by DE PCGs with significant upregulation expression in MD vs. MR. **(C)** KEGG pathway enriched by DE PCGs with strong downregulation expression in MD vs. MR. **(D)** GO entries enriched by DE PCGs with strong downregulation expression in MD vs. MR.

The upregulated molecules in the death group vs. resistance piglets were mainly involved in the TNF signaling pathway, cytokine–cytokine receptor interactions, PI3K-Akt signaling pathway, complement and coagulation cascade, focal adhesion, JAK–STAT signaling pathway, NF-κB signaling pathway ECM receptor interactions, IL-17 signaling pathway, MAPK signaling pathway, viral protein, and cytokine–cytokine receptor interactions. Additionally, the downregulated molecules were enriched to peroxisomes, ABC transporters, the signaling-angiotensin system, bile secretion, fat digestion and absorption, and tryptophan metabolism (Figure 5, (Supplementary Figures S7, S8).

### 3.5 Identification of specific expression molecules

In this study, we identified the specifically expressed PCGs, lncRNAs, and miRNAs for each group of piglets, with the expressions 36 times higher in one group than in other groups. In total, we detected 200 specific expression molecules, including 50 (28 PCGs, 16 lncRNAs, and 6 miRNAs) in the death group, 38 (21 PCGs, 9 lncRNAs, and 8 miRNAs) in the resistance group, and 112 (44 PCGs, 60 lncRNAs, and 8 miRNAs) in the control group. Using functional annotation, the specific expression molecules observed in the death group strongly correlated with environmental information pathways, such as cell–matrix adhesion, macrophage chemotaxis, positive regulation of monocyte chemotaxis, and protein kinase activator activity. The specific expression molecules of the resistance group were strongly enriched in extracellular space, and the specific expression molecules of the control group were mainly significantly enriched in metabolic pathways, such as the PPAR signaling pathway, cholesterol metabolism, and vitamin digestion and absorption.

### 3.6 Construction of competing endogenous RNA network

**Figure 6:**
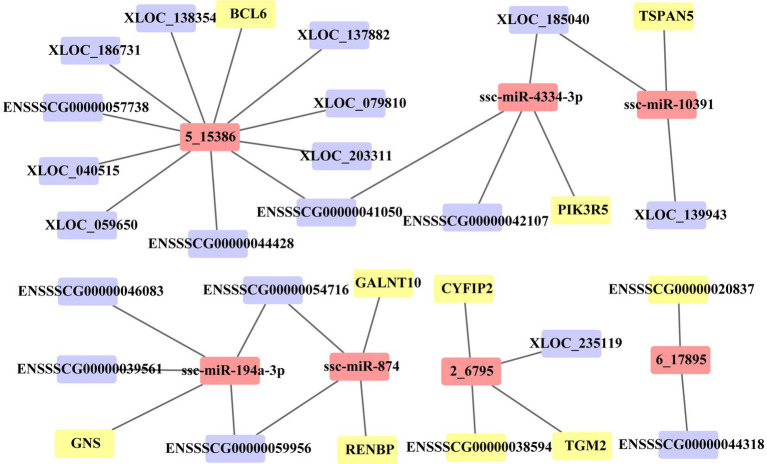
ceRNA network of 36 molecules. Three sets of differential molecules were utilized to predict the targeting interactions between PCGs, lncRNAs, and miRNAs to build the ceRNA network associated with PEDV infection in Min pigs. The picture shows pairs of relationships with correlation coefficients >0.95 and *p* < 0.05. The yellow square represents PCGs, the purple square represents lncRNAs, and the pink square represents miRNAs.

In this study, we identified the target of each DE miRNA from DE PCG and DE lncRNA transcripts. When the |correlation coefficient (cor)| between miRNA and the target was greater than 0.9, these relationships were preserved to construct ceRNA. A total of 97 PCGs, 108 lncRNAs, and 51 miRNAs are included in the ceRNA network (Supplementary Table S4). In the network, 89 (19 PCGs, 60 lncRNAs, and 13 miRNAs) and 151 (74 PCGs, 41 lncRNAs, and 36 miRNAs) molecules were, respectively, upregulated and downregulated in the death group vs. control group, and 40 (9 PCGs, 23 lncRNAs, and 8 miRNAs) and 131 (63 PCGs, 38 lncRNAs, and 30 miRNAs) molecules were, respectively, upregulated and downregulated in the resistance group vs. control group. For the death group vs. resistance group, 22 (6 PCGs and 16 lncRNAs) and 9 (2 PCGs and 7 lncRNAs) molecules were upregulated and downregulated, respectively. Through functional analysis, we found that the PCGs, lncRNAs, and miRNAs in ceRNAs were significantly enriched in 16 GO terms (*p* < 0.05) associated with immunity, such as antimicrobial peptide-mediated humoral immune response, detection of bacteria, and killing of cells from other organisms. Due to the large number of relationship pairs that cannot be fully displayed, we only visualized ceRNA pairs with |cor| > 0.95 and *p* < 0.05 in Figure 6, and the entire relationship pairs are shown in Supplementary Table S4.

### 3.7 Weighted correlation network analysis

**Figure 7:**
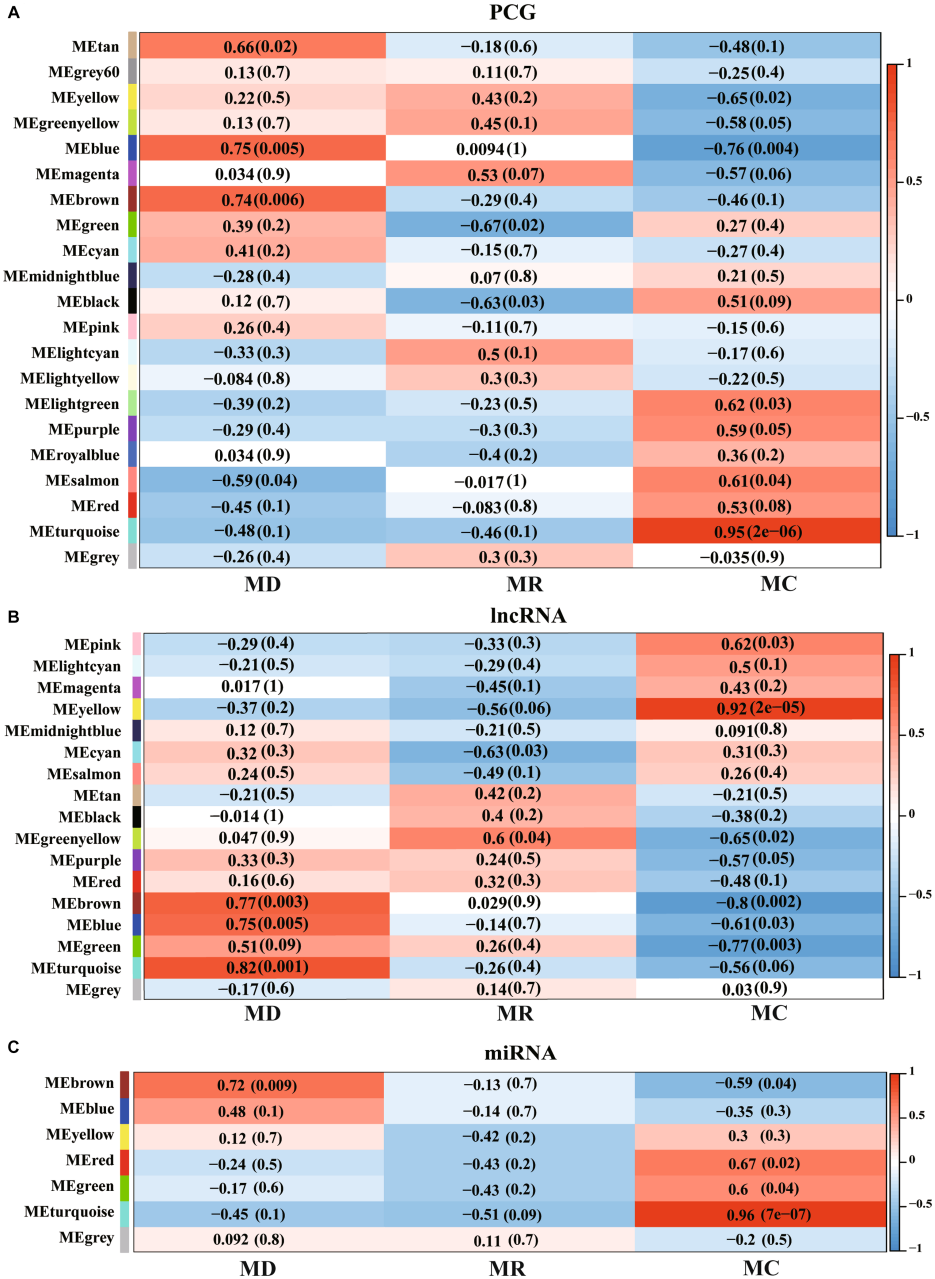
Weighted correlation network analysis (WGCNA) of PCGs, lncRNAs, and miRNAs identified in Min pigs. **(A–C)**, respectively, show the WGCNA result of expressed PCGs, lncRNAs, and miRNAs. MC: Min piglets of the control group. MD: Min piglets of the death group. MR: Min piglets of the resistance group. Numbers outside brackets represent Pearson correlation coefficients, and numbers in brackets represent *p*-values.

We performed WGCNA for PCGs, lncRNAs, and miRNAs and detected 27 significant modules (|cor| > 0.5 and *p* < 0.05), including 8 associated with the death group, 4 associated with the resistance group, and 15 associated with the control group (Figure 7). A total of 13,716 PCGs, 7,281 lncRNAs, and 563 miRNAs were identified in these 27 significant modules (Supplementary Table S4), in which, 6,177 PCGs, 2,783 lncRNAs, and 255 miRNAs were DE molecules, and 147 were the specific expressed molecules. Furthermore, we performed functional analysis of PCGs, lncRNAs, and miRNAs involved in the significant modules and found that the molecules of significant modules associated with death piglets were mainly involved in environmental information processing, such as AMPK, MAPK, HIF-1, PI3K-Akt, mTOR, and TNF. The PCGs, lncRNAs, and miRNAs involved in significant modules of resistance pigs were primarily related to environmental information processing and organism systems, including T-cell receptor, MAPK signaling pathway, focal adhesion, chemokine signaling pathway, natural killer cell-mediated cytotoxicity, Th17 cell differentiation, and NF-kappa B signaling pathway. The molecules of significant modules associated with control piglets were widely involved in metabolism, genetic information processes, human diseases, cellular processes, environmental information processes, and organismal systems.

### 3.8 Prediction of TF bound in DE PCGs

**Figure 8:**
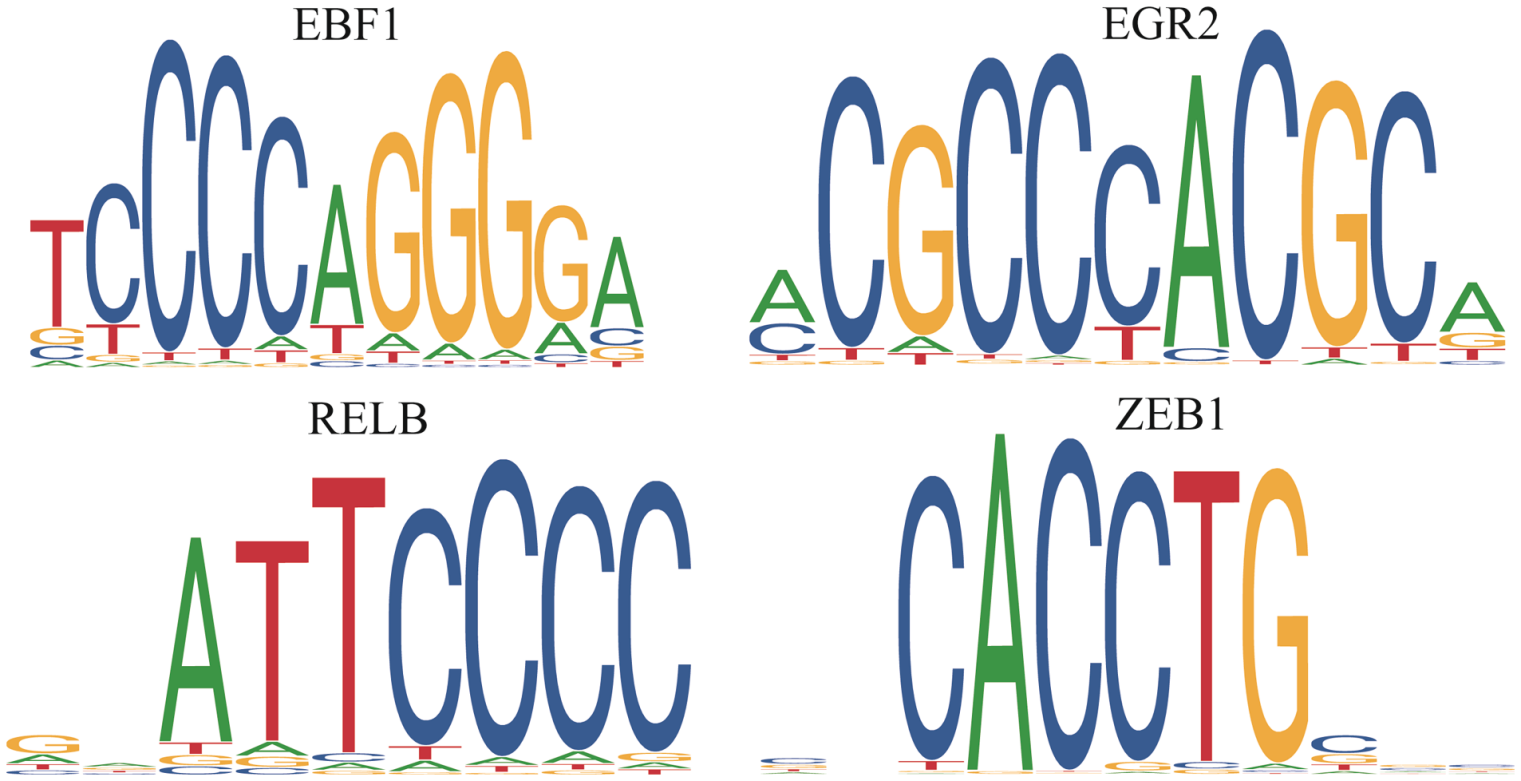
Differentially expressed transcription factors in the death piglets vs. resistance piglets.

We predicted the TFs bound with DE PCGs and found that 6,585 DE PCGs were enriched in 32 significant motifs (*E-*value < 0.05), which were annotated by 519 TFs. Among them, 53 TFs were DE PCGs, which were found to co-regulate all DE PCGs (Supplementary Table S5; Figure 8).

### 3.9 Validation

**Figure 9:**
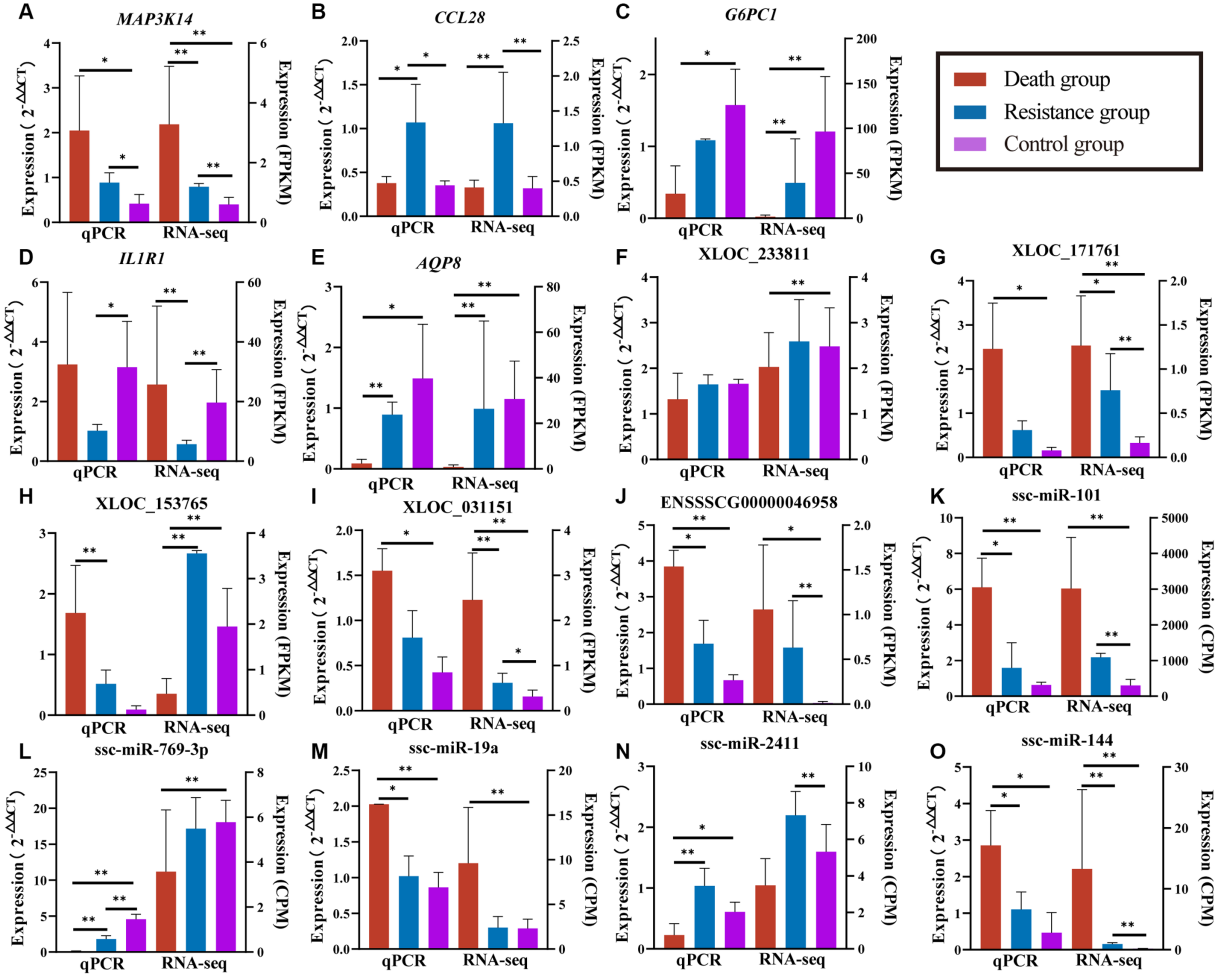
Validated results of qRT-PCR for randomly selected PCGs, lncRNAs, and miRNAs. **(A–E)** are quantitative results and FPKM values of randomly selected PCGs. **(F–J)** are quantification results and FPKM values of randomly selected lncRNAs. **(K–O)** are quantification and CPM values of randomly selected miRNAs. Quantitative results are on the left, *indicates *p*-value <0.05 and **indicates *p*-value <0.01. FPKM/CPM values of the transcriptome are on the right, *indicates adjusted *p*-value <0.05 and **indicates adjusted *p*-value <0.01.

We randomly selected five DE mRNAs, five DE lncRNAs, and five DE miRNAs for qRT-PCR to validate the quality of RNA-seq data and found that the expression patterns of PCGs, lncRNAs, and miRNAs were consistent with the RNA-seq results (Figure 9). The fold changes were different between qRT-PCR and RNA-seq data, which might be caused by the differences in sensitivity and specificity.

## 4 Discussion

The widespread prevalence of PEDV has had a serious impact on sow reproductive performance, fattening pig performance, and piglet growth (23–25). PED is an intestinal disease that severely affects the digestive and metabolic functions of pigs, and thus, the feces produced by the diseased pigs might pose a potential burden to the environment. In this study, we constructed an infection model of PEDV in Min pigs and showed the significant differences in phenotypes between the three groups. Furthermore, we performed transcriptome analysis of jejunum tissues and identified the key PCGs, lncRNAs, and miRNAs involved in the immune responses of Min pigs infected with PEDV, which provided important information on virus–host interactions for deciphering the mechanism of virus infection.

PEDV infection resulted in apoptosis and necrosis of small intestinal absorptive epithelial cells and a decrease in the ratio of villous height to crypt depth (26, 27). In this study, all the infected piglets showed significant intestinal lesions, and the death individuals were more serious than the resistant individuals. In addition, the PEDV copy number and antigen of intestinal segments in death piglets were higher than those in resistance piglets. These implied that the death of piglets infected with PEDV might be mainly due to severe intestinal damage caused by the inability to prevent the virus replication.

Differential analysis identified many differential genes broadly involved in innate immune pathways and metabolic pathways. The upregulated RNA molecules in the death vs. resistance piglets were mainly involved in innate immune pathways and signal transduction pathways. Interleukins (ILs) play critical roles in immune cell activation and regulation and inflammatory responses (28). *IL-1α* and *IL-1β* trigger a pro-inflammatory response upon binding to IL-1R1 receptor complexes (29). Upregulated expression of *IL-11* enhanced the anti-apoptotic ability of epithelial cells against PEDV infection (30), and *IL17RA* mediated the biological functions of most IL17 family members, primarily mediating pro-inflammatory and anti-inflammatory responses (31). In this study, *IL11, IL17C, IL17D, IL17RA, IL1A, IL1R1, IL1R2, IL1RAP, IL20RA, IL20RB, IL22, IL22RA2, IL26, IL27, IL29, IL2RB, IL2RG, IL4I1, IL4R,* and *IL6* significantly had upregulated expression in the infected group, which might be associated with the inhibition of viral replication. Chemokines played an important role in linking innate and acquired immune responses, which suppressed viral infections and inflammatory responses (32). Driven by the chemokine *CCL2* secreted by intestinal epithelial cells infected with PEDV, the number of lymphocytes entering the intestine increased (33). *CXCL9* secreted by PEDV-infected IPEC-J2 cells significantly regulated the number of T-cell subpopulations in the peripheral blood cells of piglets, and high expression of *CXCL13* significantly increased the number of B-cell migrations (34). The synergistic role of *CCL25* and *CCL28* in translocating IgA cells into the intestine had been demonstrated in mice, and pigs immunized with *CCL25/CCL28* adjuvant virus-like particles showed excellent immune protection against PEDV (26, 35). In this study, *CXCL8, CXCL2, CXCR6, CXCL9, CXCR4, CXCL8, CXCR6, CXCR4, CX3CL1, CCL19, CCL4, CCL21, CCL26, CCL28, and CCL11* showed higher expressions in the infected group than that in the control group, while *CX3CR1, CXCL14, CCL5, and CCL16* had lower expressions in the infected group than that in the control group. These suggested that many chemokines played active roles in host resistance to PEDV, and PEDV might ensure self-replication by inhibiting the expression of chemokines. The FoxO signaling pathway regulated cell cycle regulation, apoptosis, autophagy, oxidative stress, DNA repair, and immune regulation and played an important role in host–virus interactions and innate immunity (36). In this study, *IRS1/IRS2* might activate *PIK3CB/PIK3CD* to further activate *AKT2* leading to *FOXO* phosphorylation, which inhibited the expression of *BCL6, KLF2,* and *G6PC3* and led to the inhibition of immune regulation as well as glycolysis/glucose metabolism pathways. Th17 cells secreted *IL-17*, which promoted the proliferation of intestinal epithelial cells, increased the expression of the polyimmunoglobulin receptor (pIgR), and promoted intestinal IgA and a variety of antimicrobial peptides (37). In this study, *IL17C* bindings to *IL17RA* might activate *FOS/FOSB/RELA/NFKBIA* and produce *IL1β, TNFα, IL6, IFNγ, CXCL8, CXCL2, and CCL2* and other chemokines to help host resistance to PEDV. Viruses have the ability to manipulate some signal transduction pathways in host cells to promote their survival (38). Activation of AMPK played an important role in mediating PEDV-induced autophagy and supported viral replication (39). In this study, *STRADA* involved in the AMPK signaling pathway was significantly upregulated in the infected group, which has the function of aiding viral replication. The ErbB family is the human epidermal growth factor (EGF) receptor (40), and the EGFR gene promotes PEDV infection by impairing the antiviral activity of type I interferon (41). In addition, *ABL1, ABL2, AREG, CAMK2B, CDKN1A, EGFR, EIF4EBP1, ELK1, EREG, MAP2K7, MYC, NRG1,* and *PAK4* upregulated in the infected group were involved in the ErbB signaling pathway, which might be involved in viral replication. The upregulated RNA molecules in the control group were mainly involved in metabolic pathways. Colonic bacteria use amino acids as substrates and produce ammonia, short-chain fatty acids (acetate, propionate, and butyrate), and branched-chain fatty acids (valerate, isobutyrate, and isovalerate), among others, and these bacterial metabolites have been shown to influence the physiological functions of epithelial cells by affecting epithelial cell signaling pathways and modulating the host mucosal immune system (42, 43). In this study, *ACY1, ALDH18A1, ALDOB, ARG1, ASL, ASS1, CPS1, CS, GOT1, IDH1, MAT2B, MTR, OTC, PFKP, PSAT1, PSPH, SDSL,* and *TPI1* are involved in pathways such as arginine and proline metabolism, which were downregulated in the infected group probably due to PEDV inhibition, therefore not providing protection to the host. The intestinal tryptophan metabolite indole-3-carboxaldehyde (3-IAld) showed inhibitory activity against dextran sodium sulfate (DSS)-induced UC mice by targeting the TLR4/NF-κB/p38 signaling pathway. The compound effectively protected against colonic length shortening and DSS-induced colonic injury, and significantly reduced the severity of inflammation. In addition, 3-IAld could upregulate the expression of ZO-1 and occludin *in vivo* and *in vitro* (44). *ACAT1, AOC1, AOX1, CAT, IDO2, KMO, KYAT1, KYAT3, MAOB,* and *TPH2* involved in tryptophan metabolism were downregulated in the infected group in this study and may not protect the jejunum from injury. The fatty acid degradation pathway was downregulated in the late stage of pseudorabies virus infection, which may accumulate fatty acids synthesized by the viral envelope (45). In this study, *ACAA1, ACAA2, ACADL, ACADM, ACADSB, ACADVL, ACAT1, ACSL3, ACSL5, ALDH1B1, CPT1A, CPT2, ECI1, EHHADH, HADHA*, and *HADHB* involved in the fatty acid degradation pathway in the infected group were downregulated, which may provide conditions for viral replication.

CeRNAs were the key regulators of many biological and disease processes, such as cell cycle, tumor suppression, and cancer development (46). In this study, we created the ceRNA regulatory network, comprising 51 miRNAs, 110 lncRNAs, and 99 PCGs, which were mainly associated with cell proliferation and the ability to overcome viruses (43). The K+ and Ca++ channels were required for intestinal homeostasis (47, 48), and the assembly of PED viruses occurred through the outgrowth of intracellular membranes, such as the endoplasmic reticulum and Golgi apparatus (3). miR-194 was highly expressed in the piglet small intestine, which played an important role in intestinal epithelial maturation (49, 50). Serum miR-194b-5p might be a potential biomarker of small intestinal damage in weaned piglets (51). In the present study, these 51 miRNAs, 110 lncRNAs, and 99 PCGs were proposed as candidate molecules for the immune response to PEDV infection in Min pigs.

TFs, as “master regulators” and “selector genes,” had the functions of controlling processes of cell developmental patterns and immune responses (52, 53). In this study, 53 DE TFs were identified, which regulated 6,585 DE PCGs. *EGR1* inhibited PEDV replication by directly binding to the IFN-regulated antiviral (IRAV) promoter and upregulating IRAV expression (54). In the present study, *EGR1, EGR2,* and *EGR3* upregulating in the death and resistance piglets, might help inhibit PEDV replication in organisms. FOXC1 and FOXC2 were required for intestinal regeneration by stimulating paracrine CXCL12 and Wnt signaling (55), and FOXP1 downregulation inhibited G1/S phase cell cycle arrest by inducing the proliferation of hepatocellular carcinoma (56). In this study, six members of the FOX gene family were identified, of which, *FOXA3, FOXC1, FOXC2, FOXF1,* and *FOXJ3* upregulating in the resistance or death piglets might resist PEDV by promoting intestinal regeneration. FOXP1 was upregulated in death piglets and might aid viral replication. *RELA* and *RELB* are key TFs of the NFκB pathway, and their reduced expression leads to dysregulation of the NFκB pathway, resulting in severe autoimmune diseases and reduced immunity (57). *RELA* and *RELB* were upregulated in the death and resistance piglets and might help the host to resist PEDV by activating the NFκB pathway. KLFs are a family of TFs that control a wide range of cellular processes in different cells or tissues. *KLF1-17* is involved in many biological processes such as proliferation, migration, differentiation, inflammation, and pluripotency (58, 59). *KLF9* had been identified as a diagnostic marker associated with ulcerative colitis (60). KLF16 inhibited PEDV replication through the type I IFN signaling pathway (60). In this study, eight TFs, namely, *KLF13, KLF15, KLF16, KLF2, KLF4, KLF5, KLF6,* and *KLF9*, were predicted to play a positive role in helping the organism to resist PEDV. In summary, the 53 TFs identified as important follow-up molecules that help the host resist PEDV need to be further validated.

## 5 Conclusion

In summary, this study provided a piglet model infected with PEDV and showed the expression profiling of mRNAs, lncRNAs, and miRNAs in jejunal tissues from control, resistance, and death groups. In addition, we detected 200 specific expressed molecules and 53 key TFs for Min piglets and constructed the ceRNA regulatory networks including 51 DE miRNAs, 97 DE PCGs, and 108 DE lncRNAs. These results deepened the understanding of the interaction between the host and PEDV.

## Data availability statement

The RNA-seq data used in this study had been submitted to the NCBI Sequence Read Archive (SRA) under accession PRJNA842216.

## Ethics statement

The animal studies were approved by the Animal Ethics Committee of the Institute of Animal Science, Chinese Academy of Agricultural Sciences. The studies were conducted in accordance with the local legislation and institutional requirements. Written informed consent was obtained from the owners for the participation of their animals in this study.

## Author contributions

HL: Formal analysis, Methodology, Software, Writing – original draft. CZ: Supervision, Writing – review & editing. MZ: Resources, Supervision, Writing – review & editing. NY: Resources, Supervision, Writing – review & editing. XH: Methodology, Writing – review & editing. JX: Supervision, Writing – review & editing. LW: Conceptualization, Data curation, Funding acquisition, Investigation, Methodology, Project administration, Resources, Supervision, Writing – review & editing. LS: Conceptualization, Data curation, Funding acquisition, Investigation, Methodology, Project administration, Resources, Supervision, Writing – review & editing.

## Footnotes

^1^https://www.ncbi.nlm.nih.gov/genome/?term=pig

^2^https://mirbase.org/

^3^http://meme-suite.org/index.html

^4^https://asia.ensembl.org/Sus_scrofa/Info/Index?db=core

## References

[1] Adler M Murani E Brunner R Ponsuksili S Wimmers K. Transcriptomic response of porcine PBMCs to vaccination with tetanus toxoid as a model antigen. PloS One. (2013) 8:e58306. doi: 10.1371/journal.pone.0058306, PMID: 23536793 PMC3607572

[2] Lee C. Porcine epidemic diarrhea virus: an emerging and re-emerging epizootic swine virus. Virol J. (2015) 12:193. doi: 10.1186/s12985-015-0421-2, PMID: 26689811 PMC4687282

[3] Ducatelle R Coussement W Debouck P Hoorens J. Pathology of experimental CV777 coronavirus enteritis in piglets. II Electron Microscopic Study Vet Pathol. (1982) 19:57–66. PMID: 6280360 10.1177/030098588201900109

[4] Kane Y Wong G Gao GF. Animal models, zoonotic reservoirs, and cross-species transmission of emerging human-infecting coronaviruses. Annu Rev Anim Biosci. (2023) 11:1–31. doi: 10.1146/annurev-animal-020420-025011, PMID: 36790890

[5] Alonso C Goede DP Morrison RB Davies PR Rovira A Marthaler DG. Evidence of infectivity of airborne porcine epidemic diarrhea virus and detection of airborne viral RNA at long distances from infected herds. Vet Res. (2014) 45:73. doi: 10.1186/s13567-014-0073-z, PMID: 25017790 PMC4347589

[6] Bertolini F Harding JC Mote B Ladinig A Plastow GS Rothschild MF. Genomic investigation of piglet resilience following porcine epidemic diarrhea outbreaks. Anim Genet. (2017) 48:228–32. doi: 10.1111/age.12522, PMID: 27943331 PMC7159462

[7] Hu Z Li Y Du H Ren J Zheng X Wei K. Transcriptome analysis reveals modulation of the STAT family in PEDV-infected IPEC-J2 cells. BMC Genomics. (2020) 21:891. doi: 10.1186/s12864-020-07306-2, PMID: 33317444 PMC7734901

[8] Zhang H Liu Q Su W Wang J Sun Y Zhang J. Genome-wide analysis of differentially expressed genes and the modulation of PEDV infection in Vero E6 cells. Microb Pathog. (2018) 117:247–54. doi: 10.1016/j.micpath.2018.02.004, PMID: 29408315 PMC7125602

[9] Sun R Guo Y Li X Li R Shi J Tan Z. PRRSV non-structural proteins orchestrate porcine E3 ubiquitin ligase RNF122 to promote PRRSV proliferation. Viruses. (2022) 14:424. doi: 10.3390/v14020424, PMID: 35216017 PMC8874583

[10] Chen J Zhang C Zhang N Liu G. Porcine endemic diarrhea virus infection regulates long noncoding RNA expression. Virology. (2019) 527:89–97. doi: 10.1016/j.virol.2018.11.007, PMID: 30471453 PMC7112091

[11] Rieger J Pelckmann LM Drewes B. Preservation and processing of intestinal tissue for the assessment of histopathology. Methods Mol Biol. (2021) 2223:267–80. doi: 10.1007/978-1-0716-1001-5_18, PMID: 33226600

[12] Kim D Langmead B Salzberg SL. HISAT: a fast spliced aligner with low memory requirements. Nat Methods. (2015) 12:357–60. doi: 10.1038/nmeth.3317, PMID: 25751142 PMC4655817

[13] Pertea M Pertea GM Antonescu CM Chang TC Mendell JT Salzberg SL. StringTie enables improved reconstruction of a transcriptome from RNA-seq reads. Nat Biotechnol. (2015) 33:290–5. doi: 10.1038/nbt.3122, PMID: 25690850 PMC4643835

[14] Kang YJ Yang DC Kong L Hou M Meng YQ Wei L. CPC2: a fast and accurate coding potential calculator based on sequence intrinsic features. Nucleic Acids Res. (2017) 45:W12–w16. doi: 10.1093/nar/gkx428, PMID: 28521017 PMC5793834

[15] Li A Zhang J Zhou Z. PLEK: a tool for predicting long non-coding RNAs and messenger RNAs based on an improved k-mer scheme. BMC Bioinfo. (2014) 15:311. doi: 10.1186/1471-2105-15-311, PMID: 25239089 PMC4177586

[16] Sun L Luo H Bu D Zhao G Yu K Zhang C. Utilizing sequence intrinsic composition to classify protein-coding and long non-coding transcripts. Nucleic Acids Res. (2013) 41:e166. doi: 10.1093/nar/gkt646, PMID: 23892401 PMC3783192

[17] Langmead B Trapnell C Pop M Salzberg SL. Ultrafast and memory-efficient alignment of short DNA sequences to the human genome. Genome Biol. (2009) 10:R25. doi: 10.1186/gb-2009-10-3-r25, PMID: 19261174 PMC2690996

[18] Friedländer MR Mackowiak SD Li N Chen W Rajewsky N. miRDeep2 accurately identifies known and hundreds of novel microRNA genes in seven animal clades. Nucleic Acids Res. (2012) 40:37–52. doi: 10.1093/nar/gkr688, PMID: 21911355 PMC3245920

[19] Quinlan AR Hall IM. BEDTools: a flexible suite of utilities for comparing genomic features. Bioinformatics. (2010) 26:841–2. doi: 10.1093/bioinformatics/btq033, PMID: 20110278 PMC2832824

[20] John B Enright AJ Aravin A Tuschl T Sander C Marks DS. Human MicroRNA targets. PLoS Biol. (2004) 2:e363. doi: 10.1371/journal.pbio.0020363, PMID: 15502875 PMC521178

[21] Shannon P Markiel A Ozier O Baliga NS Wang JT Ramage D. Cytoscape: a software environment for integrated models of biomolecular interaction networks. Genome Res. (2003) 13:2498–504. doi: 10.1101/gr.1239303, PMID: 14597658 PMC403769

[22] Sherman BT Hao M Qiu J Jiao X Baseler MW Lane HC. DAVID: a web server for functional enrichment analysis and functional annotation of gene lists. Nucleic Acids Res. (2021) 50:W216–w221. doi: 10.1093/nar/gkac194 PMC9252805 35325185

[23] Olanratmanee EO Kunavongkrit A Tummaruk P. Impact of porcine epidemic diarrhea virus infection at different periods of pregnancy on subsequent reproductive performance in gilts and sows. Anim Reprod Sci. (2010) 122:42–51. doi: 10.1016/j.anireprosci.2010.07.004, PMID: 20727693

[24] Furutani A Kawabata T Sueyoshi M Sasaki Y. Impact of porcine epidemic diarrhea on herd and individual Berkshire sow productivity. Anim Reprod Sci. (2017) 183:1–8. doi: 10.1016/j.anireprosci.2017.06.013, PMID: 28683954 PMC7126730

[25] Alvarez J Sarradell J Morrison R Perez A. Impact of porcine epidemic diarrhea on performance of growing pigs. PloS One. (2015) 10:e0120532. doi: 10.1371/journal.pone.0120532, PMID: 25768287 PMC4359118

[26] Hsu CW Chang MH Chang HW Wu TY Chang YC. Parenterally administered porcine epidemic diarrhea virus-like particle-based vaccine formulated with CCL25/28 chemokines induces systemic and mucosal immune Protectivity in pigs. Viruses. (2020) 12:1122. doi: 10.3390/v12101122, PMID: 33023277 PMC7600258

[27] Kim Y Lee C. Porcine epidemic diarrhea virus induces caspase-independent apoptosis through activation of mitochondrial apoptosis-inducing factor. Virology. (2014) 460-461:180–93. doi: 10.1016/j.virol.2014.04.040, PMID: 25010284 PMC7127720

[28] Liu R Yin H Sun X Liu S Wang A Wu Y. Interleukin 20 receptor a expression in colorectal cancer and its clinical significance. PeerJ. (2021) 9:e12467. doi: 10.7717/peerj.12467, PMID: 34820194 PMC8603834

[29] Chan AH Schroder K. Inflammasome signaling and regulation of interleukin-1 family cytokines. J Exp Med. (2020) 217:e20190314. doi: 10.1084/jem.20190314, PMID: 31611248 PMC7037238

[30] Li Y Wu Q Jin Y Yang Q. Antiviral activity of interleukin-11 as a response to porcine epidemic diarrhea virus infection. Vet Res. (2019) 50:111. doi: 10.1186/s13567-019-0729-9, PMID: 31864417 PMC6925494

[31] Wilson SC Caveney NA Yen M Pollmann C Xiang X Jude KM. Organizing structural principles of the IL-17 ligand-receptor axis. Nature. (2022) 609:622–9. doi: 10.1038/s41586-022-05116-y, PMID: 35863378 PMC9477748

[32] Mackay CR. Chemokines: immunology's high impact factors. Nat Immunol. (2001) 2:95–101. doi: 10.1038/84298 11175800

[33] Li Y Ma Y Jin Y Peng X Wang X Zhang P. Porcine intraepithelial lymphocytes undergo migration and produce an antiviral response following intestinal virus infection. Commun Biol. (2022) 5:252. doi: 10.1038/s42003-022-03205-2, PMID: 35318455 PMC8941121

[34] Yuan C Sun L Chen L Guo H Yao Z Wang Y. Chemokines induced by PEDV infection and chemotactic effects on monocyte. T and B cells Vet Microbiol. (2022) 275:109599. doi: 10.1016/j.vetmic.2022.109599, PMID: 36335842

[35] Feng N Jaimes MC Lazarus NH Monak D Zhang C Butcher EC. Redundant role of chemokines CCL25/TECK and CCL28/MEC in IgA+ plasmablast recruitment to the intestinal lamina propria after rotavirus infection. J Immunol. (2006) 176:5749–59. doi: 10.4049/jimmunol.176.10.5749, PMID: 16670280

[36] Accili D Arden KC. FoxOs at the crossroads of cellular metabolism, differentiation, and transformation. Cells. (2004) 117:421–6. doi: 10.1016/S0092-8674(04)00452-0, PMID: 15137936

[37] Matsunaga Y Clark T Wanek AG Bitoun JP Gong Q Good M. Intestinal IL-17R signaling controls secretory IgA and oxidase balance in *Citrobacter rodentium* infection. J Immunol. (2021) 206:766–75. doi: 10.4049/jimmunol.2000591, PMID: 33431657 PMC8663204

[38] Bonjardim CA. Viral exploitation of the MEK/ERK pathway - a tale of vaccinia virus and other viruses. Virology. (2017) 507:267–75. doi: 10.1016/j.virol.2016.12.011, PMID: 28526201

[39] Wang J Kan X Li X Sun J Xu X. Porcine epidemic diarrhoea virus (PEDV) infection activates AMPK and JNK through TAK1 to induce autophagy and enhance virus replication. Virulence. (2022) 13:1697–712. doi: 10.1080/21505594.2022.2127192, PMID: 36168145 PMC9543055

[40] Patnaik SK Swaroop AK Nagarjuna P Nanjan MJ Chandrasekar MJN. Peptides for dual targeting of ErbB1 and ErbB2: Blocking EGFR cell signaling transduction pathways for Cancer chemotherapy. Curr Mol Pharmacol. (2023) 17:e240223214012. doi: 10.2174/1874467216666230224104950 36843255

[41] Yang L Xu J Guo L Guo T Zhang L Feng L. Porcine epidemic diarrhea virus-induced epidermal growth factor receptor activation impairs the antiviral activity of type I interferon. J Virol. (2018) 92:e02095-17. doi: 10.1128/JVI.02095-17, PMID: 29386292 PMC5874413

[42] Blachier F Mariotti F Huneau JF Tomé D. Effects of amino acid-derived luminal metabolites on the colonic epithelium and physiopathological consequences. Amino Acids. (2007) 33:547–62. doi: 10.1007/s00726-006-0477-9, PMID: 17146590

[43] Schaible UE Kaufmann SH. A nutritive view on the host-pathogen interplay. Trends Microbiol. (2005) 13:373–80. doi: 10.1016/j.tim.2005.06.009, PMID: 15993074

[44] Liu M Wang Y Xiang H Guo M Li S Liu M. The tryptophan metabolite Indole-3-Carboxaldehyde alleviates mice with DSS-induced ulcerative colitis by balancing amino acid metabolism, inhibiting intestinal inflammation, and improving intestinal barrier function. Molecules. (2023) 28:3704. doi: 10.3390/molecules28093704 37175112 PMC10180526

[45] Shangguan A Li J Sun Y Liu Z Zhang S. Host-virus interactions in PK-15 cells infected with pseudorabies virus Becker strain based on RNA-seq. Virus Res. (2022) 318:198829. doi: 10.1016/j.virusres.2022.198829, PMID: 35636585

[46] Sen R Ghosal S Das S Balti S Chakrabarti J. Competing endogenous RNA: the key to posttranscriptional regulation. ScientificWorldJournal. (2014) 2014:896206. doi: 10.1155/2014/896206 24672386 PMC3929601

[47] Field M. Intestinal ion transport and the pathophysiology of diarrhea. J Clin Invest. (2003) 111:931–43. doi: 10.1172/JCI200318326 12671039 PMC152597

[48] Moeser AJ Blikslager AT. Mechanisms of porcine diarrheal disease. J Am Vet Med Assoc. (2007) 231:56–67. doi: 10.2460/javma.231.1.56 17605665

[49] Sharbati S Friedländer MR Sharbati J Hoeke L Chen W Keller A. Deciphering the porcine intestinal microRNA transcriptome. BMC Genomics. (2010) 11:275. doi: 10.1186/1471-2164-11-275, PMID: 20433717 PMC2873480

[50] Tao X Xu Z. MicroRNA transcriptome in swine small intestine during weaning stress. PloS One. (2013) 8:e79343. doi: 10.1371/journal.pone.0079343, PMID: 24260202 PMC3832476

[51] Tao X Xu Z Men X. Analysis of serum microRNA expression profiles and comparison with small intestinal microRNA expression profiles in weaned piglets. PloS One. (2016) 11:e0162776. doi: 10.1371/journal.pone.0162776, PMID: 27632531 PMC5025173

[52] Chen XF Zhang YW Xu H Bu G. Transcriptional regulation and its misregulation in Alzheimer’s disease. Mol Brain. (2013) 6:44. doi: 10.1186/1756-6606-6-44, PMID: 24144318 PMC3854070

[53] Bilal R. Dynamics of gene regulatory networks in the immune system. London: University College London (2013).

[54] Wang H Kong N Jiao Y Dong S Sun D Chen X. EGR1 suppresses porcine epidemic diarrhea virus replication by regulating IRAV to degrade viral Nucleocapsid protein. J Virol. (2021) 95:e0064521. doi: 10.1128/JVI.00645-21, PMID: 34287043 PMC8428380

[55] Tan C Norden PR Yu W Liu T Ujiie N Lee SK. Endothelial FOXC1 and FOXC2 promote intestinal regeneration after ischemia-reperfusion injury. EMBO Rep. (2023) 24:e56030. doi: 10.15252/embr.202256030, PMID: 37154714 PMC10328078

[56] Wang X Sun J Cui M Zhao F Ge C Chen T. Downregulation of FOXP1 inhibits cell proliferation in hepatocellular carcinoma by inducing G1/S phase cell cycle arrest. Int J Mol Sci. (2016) 17:1501. doi: 10.3390/ijms17091501, PMID: 27618020 PMC5037778

[57] Sharfe N Dalal I Naghdi Z Lefaudeux D Vong L Dadi H. NFκB pathway dysregulation due to reduced RelB expression leads to severe autoimmune disorders and declining immunity. J Autoimmun. (2022) 137:102946. doi: 10.1016/j.jaut.2022.102946 36402602 PMC10861023

[58] McConnell BB Yang VW. Mammalian Krüppel-like factors in health and diseases. Physiol Rev. (2010) 90:1337–81. doi: 10.1152/physrev.00058.2009, PMID: 20959618 PMC2975554

[59] Tetreault MP Yang Y Katz JP. Kruppel-like factors in cancer. Nat Rev Cancer. (2013) 13:701–13. doi: 10.1038/nrc3582 24060862

[60] Dong S Kong N Shen H Li Y Qin W Zhai H. KLF16 inhibits PEDV replication by activating the type I IFN signaling pathway. Vet Microbiol. (2022) 274:109577. doi: 10.1016/j.vetmic.2022.109577, PMID: 36215773

