# Supplementary material for: Transcriptomics yields valuable information regarding the response mechanisms of Chinese Min pigs infected with PEDV
Source: Front Vet Sci. 2023 Dec 11;10:1295723. doi: 10.3389/fvets.2023.1295723 (PMC10773921; doi:10.3389/fvets.2023.1295723)
Supplement: Supplementary file 5 [file Image_5.pdf]

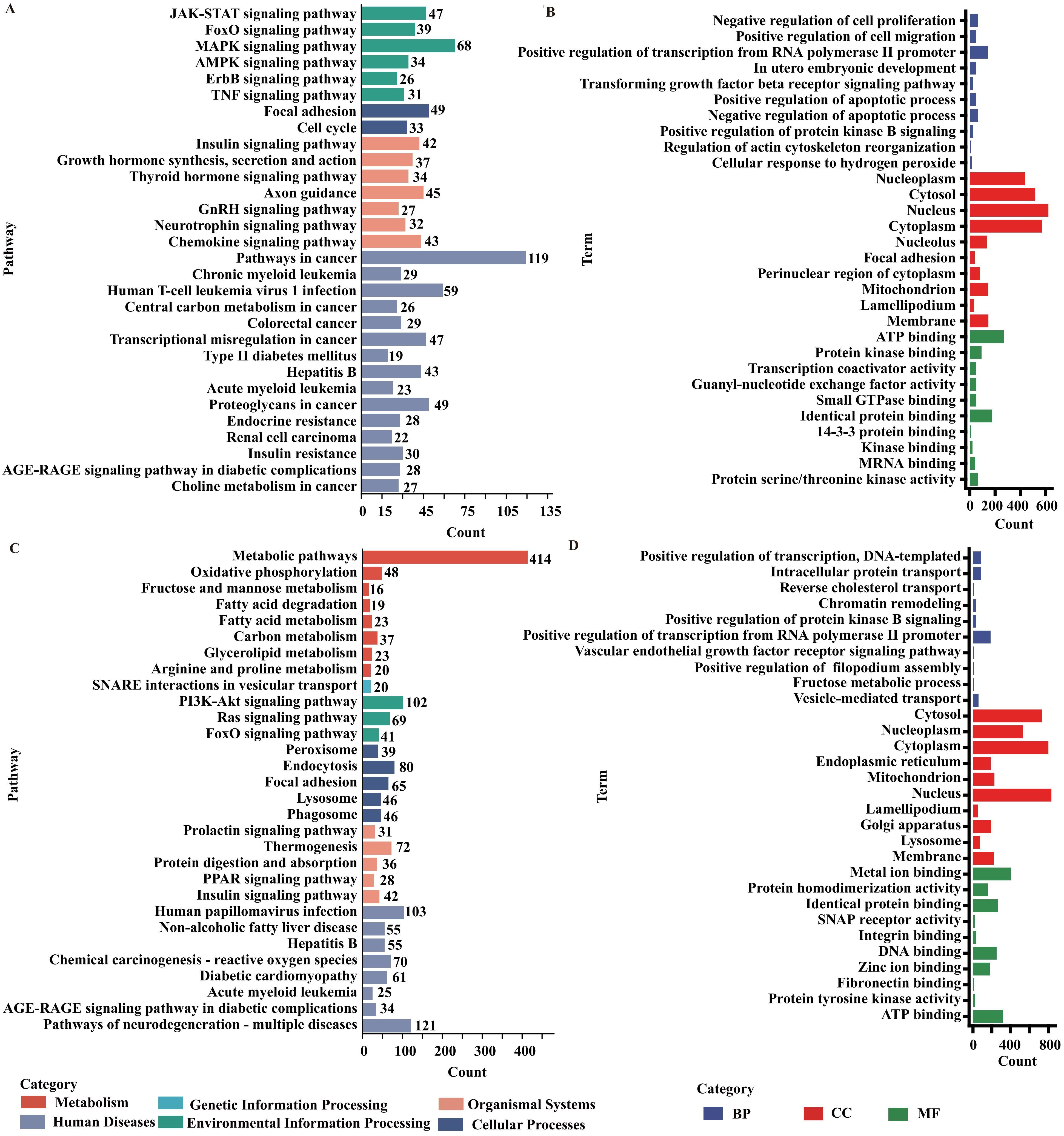

Figure S5. The enrichments of DE lncRNAs in Min resistance (MR) vs. Min control (MC) groups. (A) KEGG pathway enriched by DE lncRNAs with significant up-regulation expression in MR vs. MC. **(B)** GO entries enriched by DE lncRNAs with significant up-regulation expression in MR vs. MC. **(C)** KEGG pathway enriched by DE lncRNAs with strong down-regulation expression in MR vs. MC. **(D)** GO entries enriched by DE lncRNAs with strong down-regulation expression in MR vs. MC.
