# Supplementary material for: Transcriptomics yields valuable information regarding the response mechanisms of Chinese Min pigs infected with PEDV
Source: Front Vet Sci. 2023 Dec 11;10:1295723. doi: 10.3389/fvets.2023.1295723 (PMC10773921; doi:10.3389/fvets.2023.1295723)
Supplement: Supplementary file 7 [file Image_7.pdf]

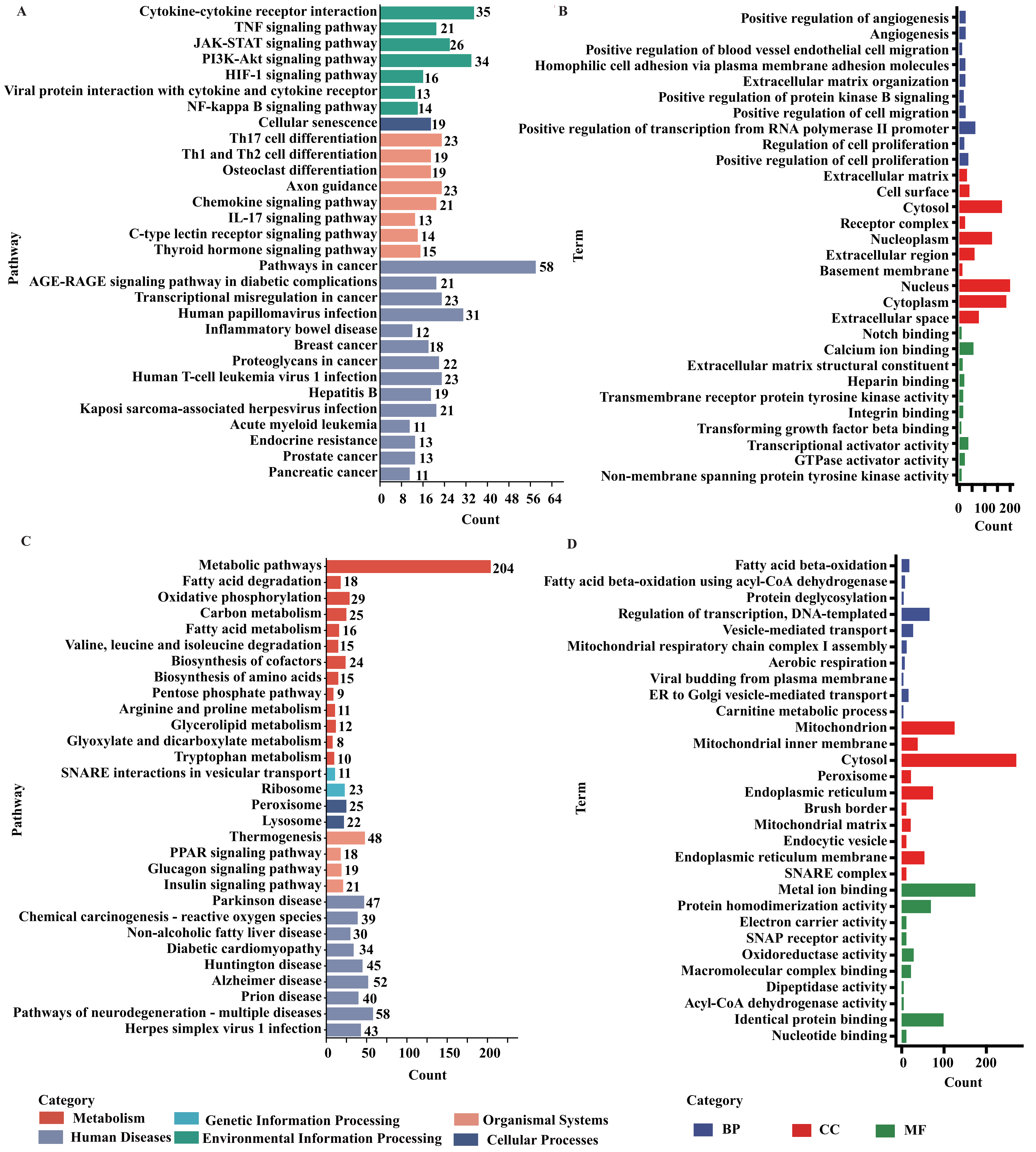

Figure S7. The enrichments of DE lncRNAs in Min dead (MD) vs. Min resistance (MR) groups. (A) KEGG pathway enriched by DE lncRNAs with significant up-regulation expression in MD vs. MR. **(B)** GO entries enriched by DE lncRNAs with significant up-regulation expression in MD vs. MR. **(C)** KEGG pathway enriched by DE lncRNAs with strong down-regulation expression in MD vs. MR. **(D)** GO entries enriched by DE lncRNAs with strong down-regulation expression in MD vs. MR.
