# Supplementary material for: Transcriptomics yields valuable information regarding the response mechanisms of Chinese Min pigs infected with PEDV
Source: Front Vet Sci. 2023 Dec 11;10:1295723. doi: 10.3389/fvets.2023.1295723 (PMC10773921; doi:10.3389/fvets.2023.1295723)
Supplement: Supplementary file 8 [file Image_8.pdf]

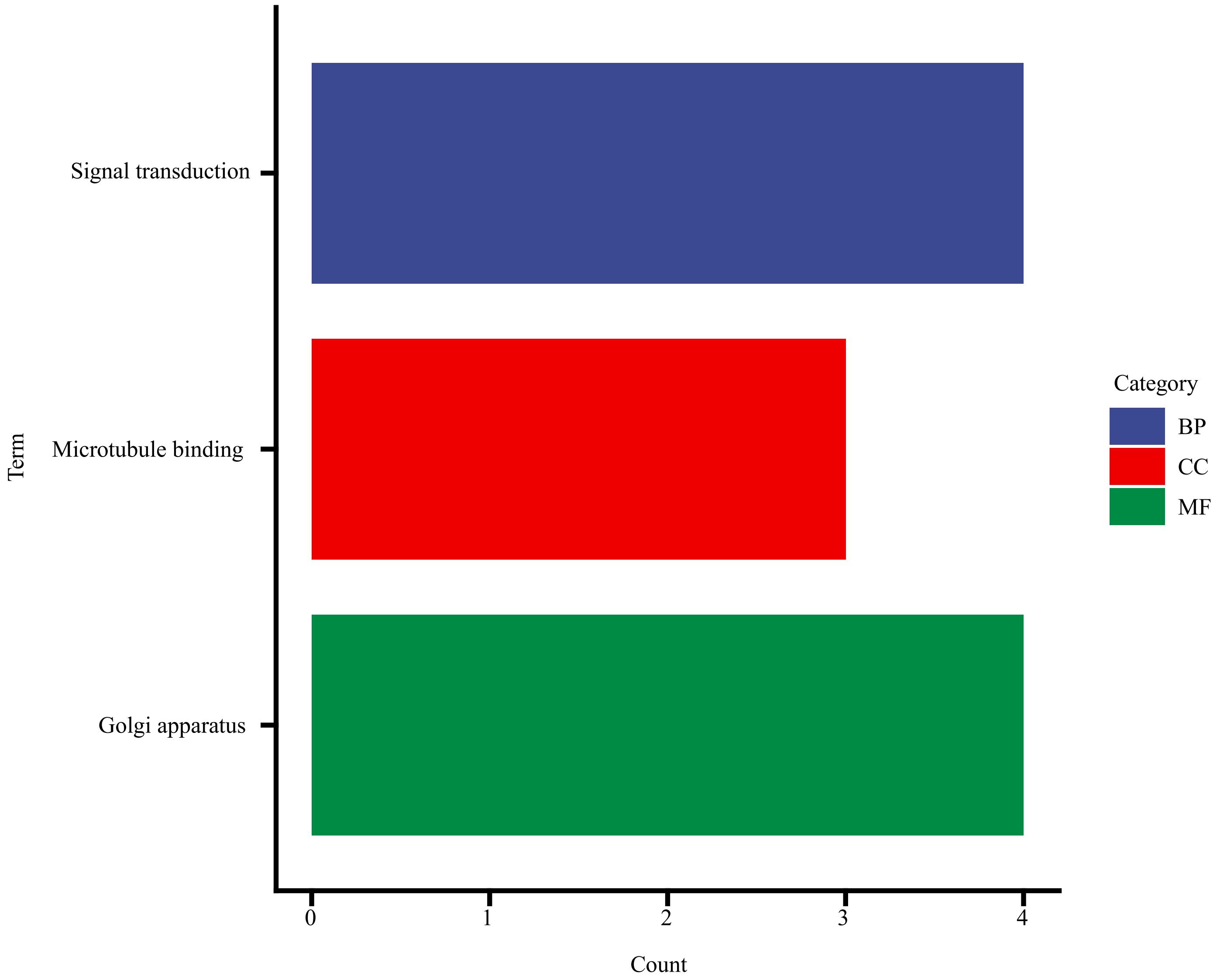

Figure S8. The enrichments of DE miRNAs in Min dead (MD) vs. Min resistance (MR) groups. GO entries enriched by DE miRNAs with strong down-regulation expression in MD vs. MR.
